# The NDR/LATS Family Kinase Cbk1 Directly Controls Transcriptional Asymmetry 

**DOI:** 10.1371/journal.pbio.0060203

**Published:** 2008-08-19

**Authors:** Emily Mazanka, Jess Alexander, Brian J Yeh, Patrick Charoenpong, Drew M Lowery, Michael Yaffe, Eric L Weiss

**Affiliations:** 1 Department of Biochemistry, Molecular Biology, and Cell Biology, Northwestern University, Evanston, Illinois, United States of America; 2 Department of Biology and Biological Engineering, Massachusetts Institute of Technology, Cambridge, Massachusetts, United States of America; Dana-Farber Cancer Institute, United States of America

## Abstract

Cell fate can be determined by asymmetric segregation of gene expression regulators. In the budding yeast Saccharomyces cerevisiae, the transcription factor Ace2 accumulates specifically in the daughter cell nucleus, where it drives transcription of genes that are not expressed in the mother cell. The NDR/LATS family protein kinase Cbk1 is required for Ace2 segregation and function. Using peptide scanning arrays, we determined Cbk1′s phosphorylation consensus motif, the first such unbiased approach for an enzyme of this family, showing that it is a basophilic kinase with an unusual preference for histidine −5 to the phosphorylation site. We found that Cbk1 phosphorylates such sites in Ace2, and that these modifications are critical for Ace2′s partitioning and function. Using proteins marked with GFP variants, we found that Ace2 moves from isotropic distribution to the daughter cell nuclear localization, well before cytokinesis, and that the nucleus must enter the daughter cell for Ace2 accumulation to occur. We found that Cbk1, unlike Ace2, is restricted to the daughter cell. Using both in vivo and in vitro assays, we found that two critical Cbk1 phosphorylations block Ace2′s interaction with nuclear export machinery, while a third distal modification most likely acts to increase the transcription factor's activity. Our findings show that Cbk1 directly controls Ace2, regulating the transcription factor's activity and interaction with nuclear export machinery through three phosphorylation sites. Furthermore, Cbk1 exhibits a novel specificity that is likely conserved among related kinases from yeast to metazoans. Cbk1 is functionally restricted to the daughter cell, and cannot diffuse from the daughter to the mother. In addition to providing a mechanism for Ace2 segregation, these findings show that an isotropically distributed cell fate determinant can be asymmetrically partitioned in cytoplasmically contiguous cells through spatial segregation of a regulating protein kinase.

## Author Summary

Cells can differentiate by segregating molecules that direct expression of specific sets of genes to one of the two cells produced by division. This generally occurs by direct mechanical movement or asymmetric anchoring of these molecules, which act after division to influence gene expression. In this study, we define a different mechanism by which the budding yeast transcription regulator Ace2 is asymmetrically partitioned. We show that Ace2 moves from uniform distribution to strong accumulation in the daughter nucleus while mother and daughter cells are still connected, and that the enzyme Cbk1 directly controls this segregation by attaching phosphate to specific sites on Ace2. We also demonstrate that Cbk1 is restricted to the daughter cell. Using both biochemical and live-cell experiments, we show that the Cbk1-mediated modifications activate Ace2 and block its interaction with nuclear export machinery, trapping it in the daughter cell nucleus. In addition to demonstrating Cbk1′s remarkable biochemical similarity to related enzymes in multicellular organisms, our analysis shows that a uniformly distributed regulator of gene expression can be made asymmetrically active in connected cells through the direct action of a localized modifying enzyme.

## Abstract 3

A conserved protein kinase, Cbk-1, produces different gene expression programs in cytoplasmically connected cells by directly blocking nuclear export of the transcription factor Ace2.

## Introduction

Cells can adopt divergent fates upon division by unequally distributing molecules or structures that direct distinct gene expression programs. The genesis of this asymmetry rests on the cell's underlying architecture, and can involve segregation of mRNAs, transcription factors, and cell surface receptors [1,2]. Unquestionably critical for metazoan development, asymmetric gene expression is also important in unicellular eukaryotes. In the budding yeast Saccharomyces cerevisiae, for example, unequal partitioning of specific transcription factors causes mother and daughter cells to express different genes late in division [3–7].

Asymmetry of intracellular cell fate determinants requires their physical segregation as well as a mechanism to ensure that they do not act before the differentiating cells are functionally separated. In a number of well-characterized cases, transcriptional regulators are directly partitioned by cytoskeleton-associated machinery.

In Drosophila melanogaster, differentiation of neuroblasts and ganglion mother cells (GMCs) is achieved through asymmetric segregation of the transcription factor Prospero's protein and mRNA, in association with the adaptor proteins Miranda and Staufen [8–10]. Miranda's segregation to the cortex of the presumptive GMC involves the actin cytoskeleton and the opposing activities of myosin VI and myosin II; this is mitotically regulated by the anaphase-promoting complex/cyclosome [11]. In the next cell cycle, Prospero translocates to the GMC nucleus, where it regulates transcription of GMC-specific genes. In budding yeast, daughter cells are prevented from switching mating types by the asymmetrically segregated transcriptional repressor Ash1. This partitioning also depends on the actin cytoskeleton: *ASH1* mRNA is transported by a class V myosin to the bud tip during mitosis and tethered to the daughter cell cortex. It remains there until the beginning of the next cell cycle, whereupon it is translated to produce the Ash1 repressor protein [4–6].

Asymmetric gene expression is also important in the last step of budding yeast cell division, but is generated by a different mechanism. Final separation of mother and daughter yeast cells requires removal of a chitin-rich septum constructed between the cells during cytokinesis [12]. Destruction of this septum occurs from the daughter side. This asymmetry is due to a daughter-specific transcriptional program driven by the transcriptional activator Ace2, which accumulates specifically in the daughter cell nucleus and induces expression of enzymes involved in septum degradation [3,13–15]. Partitioning of this transcription factor is independent of mechanisms required for *ASH1* segregation [7], and remains incompletely understood.

Ace2′s activation and daughter nucleus accumulation are coordinated with mitotic exit [7,16]. The transcription factor first localizes faintly to both mother and daughter nuclei, then accumulates to high levels exclusively in the daughter nucleus at the end of mitosis. The timing of this accumulation relative to cytokinesis is uncertain. Ace2′s nuclear import is likely blocked by mitotic cyclin-dependent kinase (CDK) phosphorylation of sites near its nuclear localization sequence (NLS) [15]: this inhibition is presumably reversed when CDK phosphorylations are removed by the phosphatase Cdc14 during mitotic exit. Ace2 nuclear export depends on the exportin Crm1/Xpo1 [7,17]. Loss of nuclear export results in symmetric Ace2 accumulation in both mother and daughter nuclei, indicating that the transcription factor is isotropically distributed in mother and daughter cells and that its asymmetry is probably not due to selective import in the daughter cell nucleus or degradation in the mother cell nucleus.

Ace2 is controlled by a conserved signaling pathway termed the Regulation of Ace2 and Morphogenesis (RAM) network [3,7,13,18]. Cells lacking RAM network function fail to separate, growing as large clusters of cells connected by the primary septum between mother and daughter cells. This separation defect is the result of failure to segregate and activate Ace2 in daughter cells and thus a lack of expression of the Ace2 target genes required for septum destruction. It is unclear how the RAM network promotes the daughter-specific segregation and activation of Ace2.

Cbk1, a protein kinase of the broadly conserved NDR/LATS family [19], is a critical component of the RAM network. Cbk1 localizes to the bud neck and daughter cell nuclei during mitosis; the kinase's nuclear localization requires Ace2 [3,7,18]. Cbk1 kinase activity is critical for Ace2 localization and activation: in cells lacking Cbk1, Ace2 localizes faintly to both mother and daughter nuclei and cannot activate transcription of its target genes [7,20]. The kinase phosphorylates an N-terminal fragment of Ace2 in vitro, suggesting a direct regulatory connection [20]. However, the identity and functional significance of Cbk1 phosphorylation sites within Ace2 are unknown.

In this study, we sought to understand how Cbk1 controls Ace2. We used an unbiased approach to elucidate the kinase's phosphorylation consensus motif and find a distinctive specificity that is likely conserved in related kinases across large evolutionary distances. This motif identified three Cbk1 phosphorylation sites within Ace2 that are crucial for the transcription factor's asymmetric distribution and function. Our in vivo and in vitro analyses of the functional significance of these sites indicate that Cbk1 phosphorylation controls Ace2 in two distinct ways: by directly blocking its interaction with nuclear export machinery and by enhancing its activity as a transcription factor. We also found that Cbk1 promotes Ace2 segregation well before cytokinesis and that the kinase is functionally partitioned to the daughter cell, allowing it to phosphorylate Ace2 and generate asymmetry from an initially isotropically distributed pool of the transcription factor.

## Results

### Ace2 Shifts from Isotropic to Asymmetric Distribution Prior to Cell Division; Nuclei Must Enter the Daughter Cell for Accumulation to Occur

**Figure 1:**
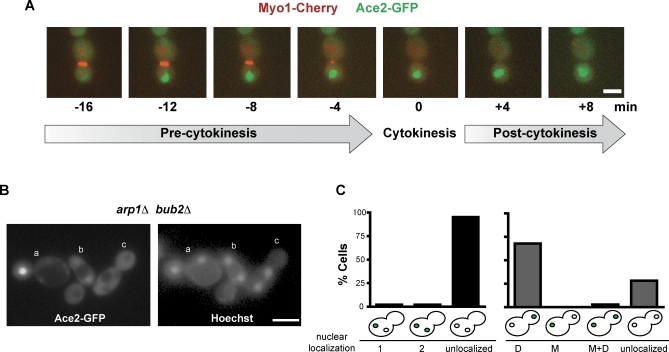
Ace2 Localization to Daughter Nuclei Occurs Prior to Cytokinesis and Requires Movement of the Nucleus into the Daughter Cell (A) Time-lapse microscopy of live cells expressing Ace2-GFP and Myo1-Cherry. Ace2-GFP localization to daughter nuclei is visible prior to disappearance of Myo1-Cherry from the bud neck. (B) Nuclear accumulation of Ace2-GFP in *arp1Δ bub2Δ* cells that fail to segregate the nucleus to the daughter. Ace2-GFP shown in left panel, and nuclear DNA stained with Hoechst shown in right panel. Ace2-GFP localizes normally in cells in which the daughter cell receives a nucleus (cell a, left panel). No nuclear accumulation of Ace2-GFP is observed in cells in which the nucleus divides entirely in the mother cell (cells b and c, left panel). Scale bar indicates 5 μm. (C) Quantification of cells shown in (B). Ace2-GFP nuclear accumulation in budded cells with two nuclei in mother cells (left graph) was scored as localized to one of the two nuclei (1), both nuclei (2), or unlocalized, *n* = 43 cells. For comparison, Ace2-GFP localization in budded cells with properly segregated nuclei (right graph) was scored as daughter nucleus only (D), mother nucleus only (M), both nuclei (M+D), or unlocalized, *n* = 53 cells.

Ace2 is initially cytoplasmically distributed in both mother and daughter cells [3,7,13], and it is therefore possible that its partitioning reflects specific regulation of the transcription factor in the daughter cell following cytokinesis. We determined whether Ace2 accumulates in the daughter cell nucleus before or after cytoplasmic separation of the dividing cells by time-lapse microscopy, comparing localization of Ace2 and the actomyosin ring component Myo1 tagged with spectrally distinct fluorescent proteins. Myo1 remains at the bud neck until cytoplasmic division is complete [21]. Before loss of Myo1 from the bud neck, small cytoplasmic proteins have been shown to freely exchange between mother and daughter cells [22]. Remarkably, we found that Ace2 localizes to daughter cell nuclei significantly prior to cytokinesis, as determined by the disappearance of Myo1 from the bud neck (Figure 1A). Thus, the transcription factor becomes asymmetrically distributed to the daughter nucleus while the mother and daughter cells are still cytoplasmically contiguous.

Ace2′s precytokinetic partitioning suggests there is an intrinsic difference between the cytoplasmic environments of the bud and the mother cell. We therefore assessed Ace2 accumulation in nuclei that divide entirely within mother cells by examining Ace2-GFP localization in an *arp1Δ bub2Δ* mutant strain. Cells lacking the dynactin component *ARP1* fail to orient the mitotic spindle properly, and nuclear division frequently occurs entirely within the mother [23]. This normally results in a mitotic checkpoint arrest; this is eliminated by deletion of the checkpoint component *BUB2* [24]. As in wild-type cells, *arp1Δ bub2Δ* cells in which nuclei migrated into daughters exhibited strong daughter-specific Ace2 nuclear accumulation (Figure 1B, cell a, and 1C, right). In contrast, Ace2 did not accumulate in nuclei of 95% of cells in which nuclear division occurred in the mother cell (Figure 1B, cells b and c, and 1C, left). Therefore, the dividing nucleus must enter the daughter cell for nuclear retention of Ace2 to occur, and the form of Ace2 that localizes to the nucleus cannot diffuse from daughter to mother cell.

### Ace2 Retention in Daughter Nuclei Does Not Require DNA Binding

**Figure 2:**
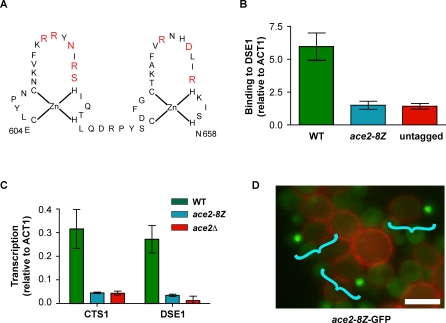
Ace2 Daughter-Specific Localization Does Not Require DNA Binding (A) Amino acid composition of Ace2 zinc finger domains are shown. Red letters denote residues predicted to participate in DNA binding. Ala substitutions were made at all eight of these sites for microscopy, ChIP, and quantitative RT-PCR analysis. (B) ChIP analysis of Ace2 binding to *DSE1* promoter shows that *ace2-8Z*-HA cannot bind its DNA target. Standard error of the mean of at least six replicates from three independent experiments is shown. WT, wild-type. (C) Quantitative RT-PCR measurement of *CTS1* and *DSE1* transcription indicates that *ace2-8Z*-GFP cannot drive expression of Ace2 target genes. (D) DNA binding-deficient mutant *ace2-8Z*-GFP is unable to promote cell separation, but localizes to daughter nuclei. Asynchronous cells were pulse labeled with rhodamine-ConA for 10 min to label the cell wall. The cells were then grown for 70 min following the removal of fluorescent lectin. Mother cells are stained in red, daughter cells are unstained. Blue brackets indicate mother–daughter pairs. Scale bars indicate 5 μm.

Ace2 accumulates strongly and equally in both mother and daughter nuclei when nuclear export is globally blocked [7]. Thus, it is unlikely that Ace2 is sequestered in the daughter cell cytoplasm or specifically degraded in the mother cell, and asymmetry may involve selective inhibition of nuclear export from the daughter nucleus [7]. One plausible mechanism for asymmetric distribution may be “anchoring” of Ace2 in the daughter nucleus by daughter-specific activation of its DNA binding capability. We therefore constructed an Ace2-GFP allele in which amino acids predicted to be essential for zinc finger-mediated DNA binding are mutated (Figure 2A). We analyzed the ability of this Ace2 allele, *ace2-8Z*-GFP, to bind its native target *DSE1* using a quantitative chromatin immunoprecipitation (ChIP) assay. This allele fails to bind its target promoter (Figure 2B), and cells carrying this *ace2-8Z*–GFP allele fail to separate and do not express Ace2 target genes (Figure 2C and 2D). To assay *ace2-8Z*-GFP localization in mother–daughter pairs that fail to separate, we briefly labeled cells with rhodamine-conjugated lectin concanavalin A, which binds stably to the cell wall, and then allowed cells to grow in the absence of the fluorescent lectin. Using this analysis, mother cells were labeled with a red fluorophore, whereas daughter cells were unlabeled, allowing for unambiguous identification of mother–daughter pairs in clumps of cells. Despite lack of functional association with its target genes*, ace2-8Z*-GFP still localized exclusively to the daughter cell nucleus (Figure 2D). Thus, Ace2 asymmetry does not arise through daughter-specific activation of the transcription factor's DNA binding.

### Cbk1 Competency to Enter Nuclei Is Daughter Cell Specific

**Figure 3:**
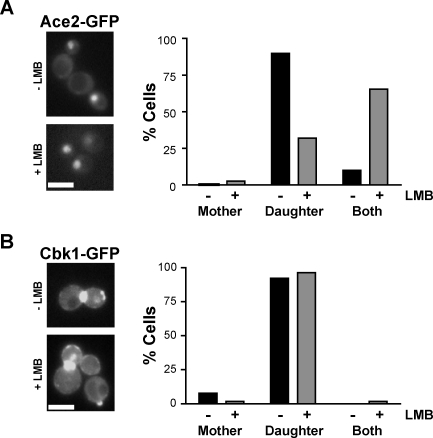
Cbk1 Is Partitioned to the Daughter Cell Mother/daughter asymmetry of Ace2-GFP (A) and Cbk1-GFP (B) following block of nuclear export. Left panels: representative cells ± 30 min treatment with leptomycin B (LMB). Scale bars indicate 5 μm. Graphs: percentage of cells exhibiting mother-only, daughter-only, or symmetric mother/daughter nuclear localization ± LMB, *n* ≥ 50 nuclei per trial.

In vivo and in vitro evidence suggests that phosphorylation by Cbk1 directly controls Ace2 partitioning and function [3,7,13,25]. Cbk1 localizes to the cortex of the growing daughter cell, as well as to the bud neck, and accumulates in the daughter nucleus [3,13,25]. Concentrating activated Cbk1 specifically in daughter cells may create a modified pool of Ace2 that could generate asymmetric localization and activation of the transcription factor. To determine whether Cbk1 that is competent to enter the nucleus is partitioned to the daughter cell, we examined the kinase's nuclear localization after nuclear export block using a sensitized Crm1 (also referred to as Xpo1) allele and leptomycin B (LMB) [26]. Unlike Ace2, which localized to both mother and daughter nuclei in LMB-treated cells (Figure 3A), Cbk1 partitioned exclusively to daughter cell nuclei in 96% of cells in which nuclear localization was visible (Figure 3B), consistent with recent findings [27]. Thus, the form of Cbk1 that is able to enter nuclei is only present in daughter cells.

### Cbk1 Is a Basophilic Kinase with a Strong Preference for Histidine at the −5 Position

**Figure 4:**
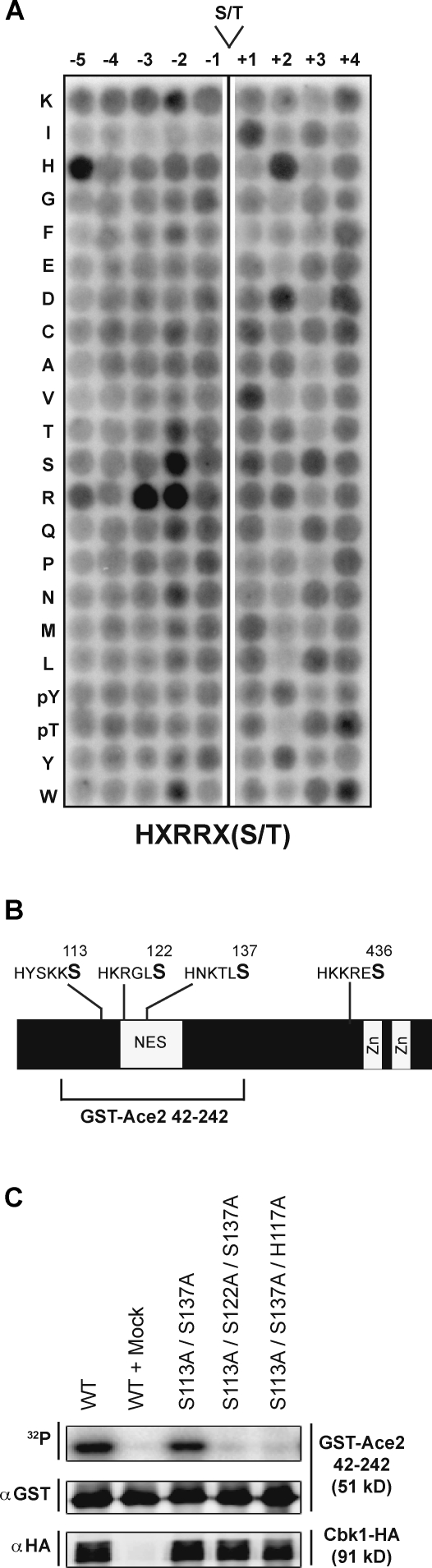
Cbk1 Exhibits Phosphorylation Site Preference for a Basic Motif with His at the −5 Position (A) Cbk1 kinase domain purified from E. coli was incubated with the indicated array of biotinylated peptide libraries in the presence of γ-^32^P-ATP, transferred to a strepavidin membrane, and kinase activity was analyzed by autoradiography. Numbers at the top of each column indicate positions relative to the phosphoacceptor S/T residue; rows contain specific amino acids in those positions. The optimal Cbk1 consensus sequence is HXRRX(S/T) as determined by quantification of the most highly phosphorylated peptide pools in the array. (B) Schematic diagram of Ace2 protein showing location of Cbk1 phosphorylation consensus sequences. (C) In vitro phosphorylation of N-terminally GST-tagged Ace2 fragments by immunoprecipitated Cbk1-HA. Combined Ala substitution of residues S113, S122, and S137 abolished ^32^P labeling of the Ace2 fragment (lane 4). His at position 117 is required for phosphorylation of S122 (lane 5). Protein levels for GST-Ace2 and Cbk1-HA were confirmed to be similar for all reactions by immunoblotting against GST and HA. WT, wild-type.

Since Cbk1 kinase activity is required for asymmetric localization and activation of Ace2, we sought to identify the sites at which Cbk1 phosphorylates Ace2. Cbk1 is related to protein kinases that prefer basic amino acids in their phosphorylation consensus motif, and prior analysis of the Cbk1-related kinase Dbf2 suggests that these kinases phosphorylate the simple basic motif Arg-X-X-(Ser/Thr), where X is any amino acid [28]. To better understand the specificity of Cbk1 and related enzymes, we determined Cbk1′s phosphorylation consensus motif using positional scanning peptide arrays [28,29]. In marked contrast to other basophilic kinases, which generally show limited specificity, Cbk1 exhibits a strong preference for the sequence His-X-Arg-Arg-X-Ser/Thr (Figure 4A). Additional selectivity is also seen for Ser in the −2 position, aliphatic amino acids in the +1 position, and for His in the +2 position. Intriguingly, the Cbk1-related *Drosophila* kinase Warts/Lats and Human LATS1 have recently been shown to phosphorylate substrates at sequences that match the Cbk1 consensus motif [30–32]. Thus, this novel and highly specific consensus motif is evidently conserved among NDR/LATS family kinases, present in eukaryotes from diverse phyla.

Previously, we demonstrated that Cbk1 phosphorylates itself both in vivo and in vitro at a conserved site in the kinase activation loop via an intramolecular reaction [20]. Cbk1′s autophosphorylation site does not match the consensus motif we have defined. This is unsurprising: activation segment autophosphorylation sites can correspond poorly to authentic consensus motifs [33], presumably because they are a special case combining high local concentration and the protein's tertiary structure.

### Cbk1 Phosphorylates Consensus Sites within Ace2

We scanned Ace2 for potential Cbk1 sites using a motif with His at −5 and the basic amino acids Lys or Arg at the −3 and/or −2 positions and identified four candidate sites: S113, S122, S137, and S436 (Figure 4B). We performed in vitro kinase reactions using an Escherichia coli expressed GST-tagged fragment of Ace2 (amino acids 42–242) that contains three of the four putative phosphorylation sites and Cbk1-HA immunoprecipitated from yeast. Larger fragments containing all four sites were prohibitively difficult to express and purify. Cbk1-HA efficiently phosphorylated this Ace2 fragment, as well as mutant Ace2 fragments in which each of the single putative phosphoacceptor serines were replaced with alanines (Figure S1). However, replacement of all three serines abolished phosphorylation, demonstrating that these constitute bona fide Cbk1 in vitro phosphorylation sites (Figure 4C). Consistent with this conclusion, recent mass spectrometric studies have shown that at least one of these sites (S122) is likely phosphorylated in vivo [34,35]. Based on these in vitro phosphorylation results, we propose that Cbk1 phosphorylates motifs with His at the −5 position and the basic amino acids Lys or Arg at either the −3 or −2 positions, concisely noted as HX(K/R)(K/R)X(S/T).

To evaluate the importance of Cbk1′s specificity for His at the −5 position, we compared in vitro phosphorylation of GST-*ace2-S113A/S137A* fragment, which contains a single phosphoacceptor site (S122), with phosphorylation of GST-*ace2-S113A/S137A/H117A*, in which His in the −5 position of the S122 site is mutated. This latter fragment was not phosphorylated, indicating that His in the −5 position is critical for Cbk1′s efficient phosphorylation of its cognate sites (Figure 4C). As discussed further below, these data are in agreement with recent findings for *Drosophila* Warts/Lats [30,31], further emphasizing the conservation of this substrate specificity among diverse eukaryotes.

### Cbk1 Phosphorylation Sites Are Critical for Ace2 Segregation and Function

**Figure 5:**
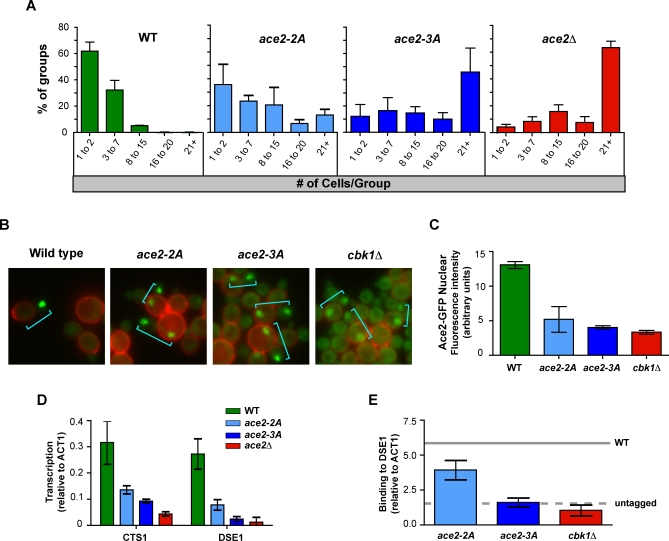
Phosphoacceptor Amino Acids S122, S137, and S436 Are Required for Proper Ace2 Localization and Function In Vivo (A) Quantification of cell separation defect in *ace2* mutant strains, shown as the percentage of groups of cells in the population in clumps of the indicated number of connected cells. The number of connected cells per group was counted in three separate trials (*n* = approximately 200 groups per trial) for wild-type (WT), *ace2-2A*, *ace2-3A*, and *ace2Δ* strains; standard deviation of the mean fraction of the population per group for the three trials is shown (analysis of other alleles, Figure S3). (B) Localization of wild-type Ace2-GFP, double-mutant *ace2-2A-GFP*, triple-mutant *ace2-3A*-GFP, and wild-type Ace2-GFP in a *cbk1Δ* strain. Mother cells were labeled with rhodamine-ConA (red); daughter cells were unlabeled. Blue brackets indicate mother–daughter pairs. Cells carrying *ace2-3A*-GFP accumulate as large clusters, and *ace2-3A*-GFP localizes to both mother and daughter nuclei. Images were all contrast enhanced to the same setting for direct comparison. Scale bar indicates 5 μm. (C) Quantification of GFP fluorescence intensity in 40 individual nuclei of large budded mother–daughter paired cells carrying *ace2-3A*-GFP, compared with *ace2-2A-GFP* and Ace2-GFP in both wild-type and *cbk1Δ* strains. Error bars indicate standard error of the mean. (D) Expression of Ace2 target genes *CTS1* and *DSE1* measured by quantitative RT-PCR demonstrated that *ace2-3A* was defective for transcriptional activity. Shown are the average values and standard deviation of three independent trials. Gene expression in all *ace2* mutant strains is shown in Figure S4. (E) Analysis of DNA binding to *DSE1* promoter shows that *ace2-3A*-HA fails to bind DNA targets, comparable to the loss of binding seen in an Ace2-HA *cbk1Δ* strain. Standard error of the mean of at least six replicates from three independent experiments is shown. Solid line indicates wild-type level of binding; dashed line denotes untagged control (as shown in Figure 2).

To determine the in vivo significance of Cbk1 phosphorylation of Ace2, we constructed a series of Ace2 mutant alleles representing all combinations of Ala substitutions at all four phosphorylation sites. For each of these alleles, we determined the abundance, localization, competence to promote cell separation, and ability to induce transcription of two Ace2 target genes (*DSE1* and *CTS1*) using quantitative real-time polymerase chain reaction (RT-PCR). For a subset of alleles, we used fluorescence microscopy to quantify the nuclear accumulation of each GFP-tagged protein. We integrated all mutant alleles at the endogenous chromosomal locus, C-terminally tagged with ether GFP or HA; all alleles were expressed at similar levels (Figure S2). To assay cell separation, we counted the number of cells in each group of cells using differential interference contrast (DIC) microscopy. Wild-type cells do not have a separation defect, and accumulated in groups of one to two cells, although larger groups sometimes formed (Figure 5A). In contrast, *ace2Δ* cells accumulated as large groups often containing 30 or more cells per group (Figure 5A), identical to the separation defect seen in *cbk1Δ* cells (Figure S3) and consistent with previous results [20]. Single Ala substitutions at the Cbk1 phosphorylation sites did not affect Ace2-GFP function, as measured by cell separation and ability to activate target transcription (Figures S3 and S4). Furthermore, they did not affect partitioning of the transcription factor to the daughter cell (unpublished data).

Two phosphorylation sites (S122 and S137) lie within Ace2′s putative nuclear export sequence (NES) [17]. We constructed a double-mutant *ace2-S122A/S137A* allele (referred to as *ace2-2A*) and found that elimination of these sites significantly affected Ace2′s partitioning to the daughter cell, yielding a substantial increase in the fraction of cells in which the transcription factor is present at low levels in both mother and daughter nuclei (Figure 5B and 5C). However, the *ace2-2A* allele conferred only an intermediate cell separation defect and an incomplete reduction of target gene expression, indicating that it supplied significant in vivo function (Figures 5A and 5D). In contrast, cells carrying a triple-mutant *ace2-S122A/S137A/S436A* (*ace2-3A*) allele failed to separate, forming large clusters of cells, and failed to activate *CTS1* and *DSE1* transcription (Figures 5A and 5D). This triple-mutant *ace2-3A*-GFP no longer accumulated specifically in daughter cell nuclei and instead mislocalized to both mother and daughter nuclei (Figure 5B). The amount of *ace2-3A*-GFP present in nuclei was significantly reduced relative to wild type and was comparable to the amount of Ace2-GFP present in nuclei of cells in a *cbk1Δ* strain (Figure 5C).

We assayed the ability of *ace2-3A*-HA to bind to its target promoters using quantitative ChIP and found that binding is severely impaired compared to the wild-type allele (Figure 5E). Intriguingly, the *ace2-2A*-HA allele, which retains the Cbk1 consensus site at Ser 436, still associates with these promoters (Figure 5E). Thus, phosphorylation of the Ser 436 site may promote or stabilize DNA interaction. Taken together, these data indicate that three Cbk1 phosphorylation sites on Ace2 are required for proper transcription factor function and segregation.

### Cbk1 Phosphorylation of Ace2′s NES Directly Blocks Interaction with Crm1

**Figure 6:**
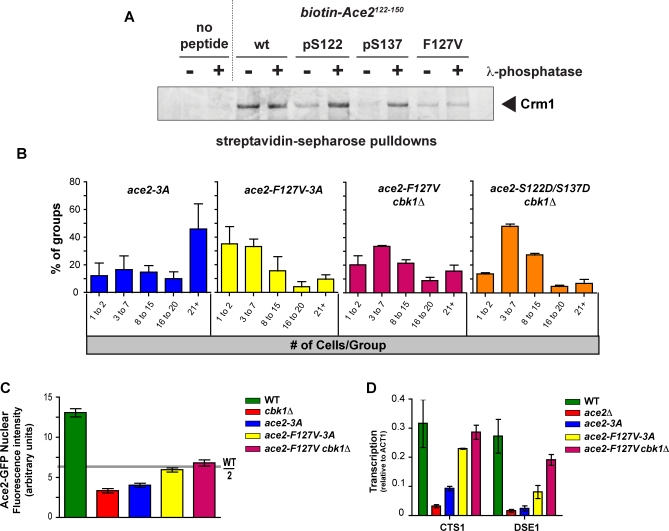
Mutation of *ace2-3A* NES Suppresses Loss of Function Phenotype (A) A biotinylated peptide encompassing residues 122–150 of Ace2 was sufficient to interact with Crm1 in an in vitro pulldown experiment. Peptides containing phosphoserine at positions 122 and 137 bound Crm1 more weakly; however, wild-type (WT) binding was restored by pretreatment of the peptides with λ-phosphatase. A F127V mutation in Ace2 also weakened the interaction with Crm1. (B) Quantification of cell separation in *ace2-F127V-3A* and *ace2-S122D/S137D* strains compared to *ace2-3A* and *ace2-F127V cbk1Δ*. *cbk1Δ* quantification is shown in Figure S3. Three independent trials of 200 groups of cells were counted and binned into different clump sizes as shown. Percentage of the population found in each clump size and standard deviations are shown. Addition of the F127V mutation suppresses the cell separation defect of *ace2-3A* cells. (C) Fluorescence intensity of each GFP-tagged Ace2 allele was quantified in approximately 40 nuclei of large budded mother–daughter pairs. *ace2-F127V-3A*-GFP shows increased nuclear accumulation compared to *ace2-3A*-GFP. Standard error of the mean is shown. (D) Quantitative RT-PCR of Ace2 target genes *CTS1* and *DSE1* is shown for each Ace2 allele. Mutation of F127V in the *ace2-3A* background increases transcriptional activity. Shown is the average of three trials with error bars denoting standard deviation.

Our findings suggest that modification of sites S122 and S137 blocks Ace2′s interaction with Crm1. Alternatively, phosphorylation of the sites may promote an intramolecular rearrangement or recruitment of another protein that antagonizes this interaction. To evaluate this directly, we first verified that amino acids 122–150 of Ace2 were sufficient to interact with purified Crm1 in an in vitro pulldown assay (Figure S5); intriguingly, this does not require Ran-GTP. To determine the effect of phosphorylation, we obtained biotinylated synthetic peptides consisting of amino acids 122–150 and incorporated phosphoserine at positions 122 or 137. Immobilizing these peptides on streptavidin-sepharose allowed us to qualitatively assess their affinity for Crm1. Ace2(122–150, pS122) bound Crm1 more weakly than unphosphorylated peptide, and the interaction was virtually abolished with Ace2(122–150, pS137) (Figure 6A). However, dephosphorylating the phosphopeptides with λ-phosphatase restored binding to both, confirming that differences in Crm1 affinity were due to the phosphoryl groups and not to differences in the efficiency of peptide immobilization. Therefore, phosphorylation of S137, and to a lesser extent S122, directly antagonizes the interaction of Ace2 with nuclear export machinery.

### Mutations within Ace2 NES Can Partially Suppress *ace2-3A*

We reasoned that replacement of Cbk1 phosphorylation sites with the acidic amino acids Asp or Glu might mimic Cbk1 phosphorylation and suppress the Ace2 loss of function seen in *cbk1Δ* cells. Substitution of S122 by Asp or Glu partially rescued cell separation in vivo (unpublished data), and substitution of both S122 and S137 to Asp significantly increased cell separation (Figure 6B). We also assayed in vitro interaction with Crm1 using an N-terminally GST-tagged fragment containing amino acids 122–150 of Ace2. We found that replacement of either S122 or S137 with either acidic amino acid (Asp or Glu) significantly reduced interaction with Crm1 (Figure S5 and unpublished data). Consistent with our in vivo results, in vitro interaction was only fully blocked by a double substitution of both S122 and S137 sites.

Mutations within the nuclear export region (F127V or G128E) allow Ace2 to accumulate in both mother and daughter nuclei and to drive transcription of target genes in the absence of Cbk1 function [25]. Biotinylated Ace2(122–150, F127V) showed very weak binding to Crm1 in vitro (and treatment with λ-phosphatase had no effect) (Figure 6A); thus, we predicted that the F127V mutation would restore activity to the *ace2-3A* allele, which exhibits defects similar to a *cbk1Δ* strain (Figure S3). Consistent with this, addition of the F127V substitution to *ace2-3A* allele restored mother–daughter separation to the level seen in an *ace2-F127V cbk1Δ* strain (Figure 6B): phenotypic suppression was incomplete in both cases. We measured the fluorescence intensity of *ace2-F127V-3A*-GFP allele in individual mother and daughter nuclei and found that it was substantially increased, to approximately half of the daughter nuclei accumulation seen in a wild-type cell (Figure 6C). Since the F127V mutation results in Ace2 that localizes to both mother and daughter nuclei evenly, we would indeed predict that the maximal accumulation of this allele in cells would only reach half that seen in a wild-type allele, in which Ace2 solely accumulates in the daughter cell nucleus. The *ace2-F127V-3A* allele also increased transcription of both the *CTS1* and *DSE1* target genes (Figure 6D). Interestingly, this rescue is considerably larger for the *CTS1* gene than the *DSE1* gene, suggesting these genes are differentially sensitive to Ace2 activity.

## Discussion

Ace2′s accumulation in daughter cell nuclei and association with target promoters is precisely linked with the end of mitosis [16,36]. This coordination likely reflects a transition from mitotic CDK phosphorylation, which blocks Ace2′s nuclear import [15,16], to postmitotic positive regulation by the NDR/LATS kinase Cbk1. The transcription factor's asymmetric partitioning, as well as its dependency on Cbk1 function, can be eliminated by treatments that block its nuclear export [7,27]. These findings show that Ace2 is initially isotropically distributed in the mother and daughter cells and that elevating its intranuclear concentration increases expression of Ace2 target genes; they suggest that the transcription factor might be regulated through control of its nucleocytoplasmic shuttling.

Our results illuminate the mechanism by which Cbk1 controls Ace2′s activity and asymmetric localization. Early in the cell cycle, Cbk1 accumulates at the cortex of the daughter cell, where it participates in bud morphogenesis. Upon mitotic exit, the kinase is then enabled to interact with and phosphorylate Ace2 in the daughter cell. We found that Cbk1 phosphorylates Ace2 at three functionally important sites, producing two distinct regulatory effects on the transcription factor. Phosphorylation of amino acids within the Ace2 NES (S122 and S137) block its interaction with the exportin Crm1 and promote its retention in the daughter nucleus; our in vitro studies exclude the possibility that phosphorylation promotes recruitment of an accessory factor or distal inhibitory domain of Ace2. This direct control of NES function is likely a general mechanism for regulation of nucleocytoplasmic shuttling: although not determined with fully purified components, phosphorylation of sites in the cyclin B1 NES likely blocks exportin binding [37]. Modification of an additional site proximal to the DNA-binding domain (S436) may play a role in enhancing transcriptional activity.

These distinct regulatory inputs appear to act in parallel to produce a more sharply defined gene expression response. Phosphorylation of Ace2′s NES allows it to accumulate to high levels in the daughter cell nucleus. These NES modifications are required for Ace2 partitioning, but they are not fully necessary for induction of Ace2-responsive genes. The *ace2-2A* allele lacking these phosphorylation sites retains the ability to induce target genes due to the presence of the S436 phosphorylation site (Figure 5D). Similarly, the S436 site is not necessary for Ace2 function when the NES phosphorylation sites are present, as seen in the *ace2-S436A* allele, indicating that accumulation of large amounts of Ace2 in the nucleus can still drive expression of target genes, albeit at a reduced level (Figure S4). Conversely, nuclear localization is not sufficient for full function: cell separation and activation of *DSE1* transcription remain partially compromised in a mutant that accumulates in both mother and daughter nuclei, but cannot be phosphorylated at S436 (*ace2-F127V-3A*). Thus, both regulatory inputs by Cbk1 phosphorylation are important for Ace2 distribution and function.

Remarkably, Cbk1-mediated accumulation of Ace2 in the daughter cell nucleus occurs significantly before cytokinesis. This regulation exemplifies a distinct system for partitioning an otherwise isotropically distributed transcription factor in cells that remain cytoplasmically contiguous. Nuclear localization of Cbk1 requires Ace2 [3,7]; therefore, we propose that Cbk1 phosphorylates Ace2 in the daughter cell cytoplasm, and the proteins enter the nucleus as a complex. The mechanism that restricts activation of Ace2 by Cbk1 to the daughter cell remains unclear. We propose two plausible models. One possibility is that a barrier at the bud neck restricts diffusion of cytoplasmic proteins between mother and daughter cells: although GFP exchanges rapidly between mother and daughter cells [22], the mobility of larger complexes has not been investigated. Therefore, Ace2 might diffuse freely between mother and daughter cells, whereas the Ace2-Cbk1 complex that forms in the daughter cell might not pass through the bud neck. Alternatively, regulatory proteins that antagonize the Ace2-Cbk1 interaction could be localized at or near the bud neck, establishing a steep activation gradient between mother and daughter cells. We previously demonstrated that phosphorylation of Cbk1′s CT motif following mitosis is not necessary for its kinase activity, but is critical for its ability to regulate Ace2 [20], and reversal of this modification by phosphatases concentrated near the bud neck could allow for spatial control of Cbk1 function. A similar effect may be achieved by dephosphorylation of Ace2 at the bud neck.

Our findings also reveal a remarkable evolutionary conservation of substrate specificity in NDR/LATS family kinases: basic phosphorylation motifs with a marked preference for His at the −5 position. Using this motif, we were able to identify critical Cbk1 phosphorylation sites in Ace2 and demonstrated that Cbk1 can phosphorylate sites in which either Lys or Arg are present at the −3 or −2 positions. The Cbk1-related *Drosophila* kinase Warts/Lats has recently been shown to phosphorylate the transcriptional coactivator Yorkie (Yki) at sequences that match the Cbk1 consensus motif to regulate nuclear localization [30,31]. Similarly, human LATS1 phosphorylates the mammalian Yki ortholog YAP at these consensus sequences [32]. In Ace2, modification directly blocks interaction with the exportin Crm1, while phosphorylation of Yki recruits a 14-3-3 protein that antagonizes nuclear import [30,31]. Thus, the yeast and metazoan kinases have inverse functional output: Cbk1 promotes nuclear accumulation of a transcription factor, whereas Warts/LATS acts to suppress it. Despite this difference, regulation of Ace2 by Cbk1 should provide important insight into regulation by this highly conserved family of protein kinases.

## Materials and Methods

### Strains and plasmids.

All strains generated and used in this study are listed in Table 1. Ace2 point mutants were created by site-directed QuikChange mutagenesis of pELW487 using Pfu Turbo (Stratagene) and integrated into ELY128 along with a C-terminal Longtine GFP::KanMX or HA::TRP1 tag [38] using two-fragment PCR. LMB-sensitive strains were created in ELY570 by integration of a C-terminal Longtine GFP::KanMX tag at the Ace2 or Cbk1 loci. ELY798 was generated by integration of a C-terminal Longtine GFP::KanMX tag at the Ace2 locus. Arp1 was deleted using the Euroscarf::LEU2 deletion plasmid [39]. All plasmids generated and used in this study are listed in Table 2. Oligos used for PCR are listed in Table 3.

**Table 1:**
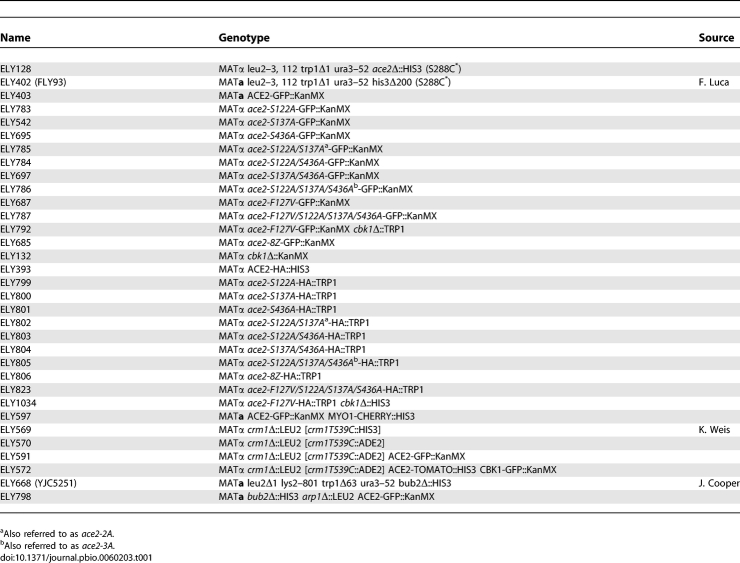
Genotypes of All Yeast Strains Used and Generated for Study

**Table 2:**
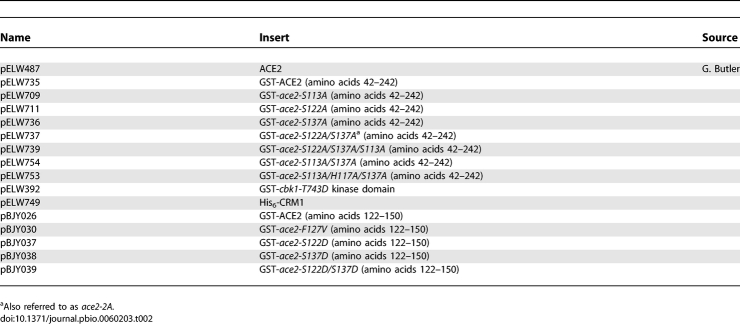
Plasmids Used and Generated in This Study

**Table 3:**
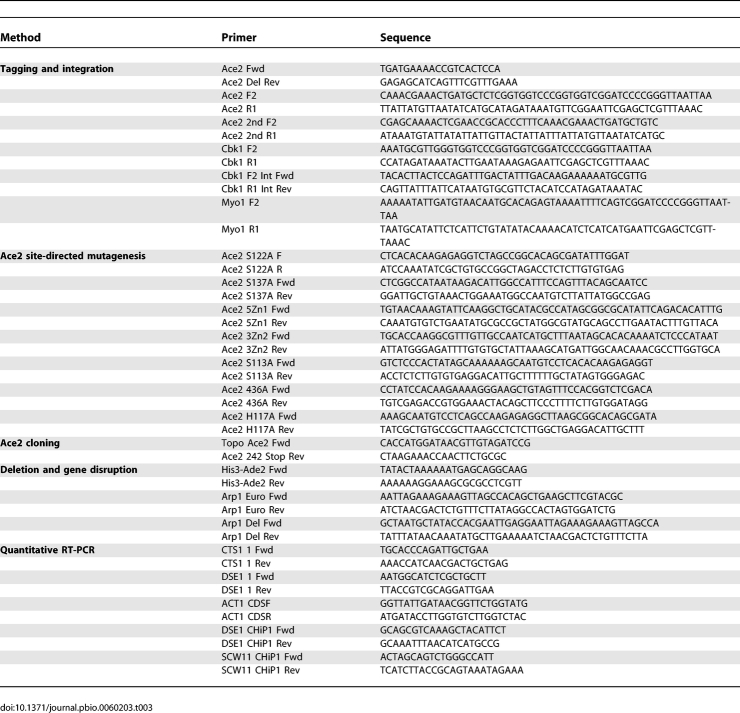
Oligos Used and Generated for This Study

### Protein purification.

BL21(DE3) expression cells containing N-terminally glutathione S-transferase (GST)-tagged Ace2 mutant fragments were grown to an optical density at 600 nm (OD_600_) = 0.7 in LB/AMP medium at 37 °C and induced with 0.5 mM IPTG at 24 °C for 4 h. Cells were spun down and pellets were frozen at −20 °C, then lysed in GST lysis buffer (40 mM Tris [pH 8], 150 mM NaCl, 0.5% Triton X-100, 1 μg/ml pepstatin, 0.5 mM leupeptin, and 1 mM PMSF) with 0.1 mg/ml lysozyme and DNase treated. Lysates were cleared by 20 min centrifugation at 10,000 rpm. Glutathione-sepharose beads (Amersham) prewashed with GST lysis buffer were added to lysates and incubated at 4 °C for 3 h rotating. Beads were washed 2× 10 ml with GST lysis buffer followed by 2× 10 ml with GST wash buffer (50 mM Tris [pH 9], 200 mM NaCl) in a column. Beads were incubated in GST elution buffer (GST wash buffer + 25 mM glutathione) for 30 min and eluted by gravity drip. Protein concentrations were measured by Bradford assay (Bio-Rad), using BSA to generate a standard curve. Glycerol was added to a final concentration of 10%, and proteins were stored at −80 °C.

N-terminally GST-tagged Cbk1-T743D kinase domain was expressed and purified as described above with an additional 1 h incubation at 4 °C postlysis with 2 mM ATP and 10 mM MgSO_4_ to remove the 70 kDa copurifying contaminating band. Proteins were concentrated in an Amicon Ultra Centricon 30 kDa MWCO, exchanging into storage buffer (20 mM Tris [pH 8], 150 mM NaCl, 10% glycerol).

Crm1 was cloned into pET100 (Invitrogen), expressed as a hexahistidine fusion in BL21(DE3)RIL, and purified by chromatography on Ni-NTA resin (Qiagen). Purified Crm1 was dialyzed into PBS/KMD buffer (25 mM sodium phosphate, 150 mM NaCl, 3 mM KCl, 1 mM MgCl_2_, 2 mM DTT), flash frozen in liquid nitrogen, and stored at −80 °C. Wild-type and mutant Ace2(122–150) were cloned into pGEX-4T1 (Amersham) and expressed as GST fusions in BL21(DE3)RIL.

### Cbk1 phosphorylation motif determination by peptide library array screening.

Positional scanning oriented-peptide library screening was performed as previously described [29]. Briefly, solution-phase kinase reactions were performed in parallel on 198 separate biotinylated, partially degenerate, oriented peptide libraries (Anaspec) arrayed in a 384-well microtiter plate in a 22 row × nine column format. Each peptide library contains an N-terminal biotin tag, a 50:50 mix of serine and threonine at the orienting phosphoacceptor residue, a single second fixed amino acid located between the −5 and +4 position, and a mixture of amino acids at all other positions. Individual libraries contain any of the 20 natural amino acids as well as phosphothreonine and phosphotyrosine in the second fixed position, corresponding to the 22 rows. Scanning across the columns in the array moves the position of the fixed amino acid from −5 to +4 relative to the fixed phosphoacceptor residue. Kinase reactions were performed at 30 °C for 6 h in a total volume of 16 μl containing 7.92 μg of recombinant purified Cbk1 kinase domain, 31.25 μM peptide library, 100 μM ATP, and 200 μCi of γ-^32^P-ATP, in 150 mM NaCl, 10 mM MnCl_2_, 1 mM Tris (2-carboxyethyl) phosphine (TCEP), and 50 mM Tris (pH 7.5). Following incubation, 2 μl of each reaction were simultaneously transferred to a streptavidin-coated membrane (Promega SAM^2^ biotin capture membrane) using a 384-slot pin replicator (VP Scientific). The membrane was washed three times with 140 mM NaCl, 0.1% SDS, 10 mM Tris (pH 7.4), three times with 2 M NaCl, twice with 2 M NaCl containing 1% H_3_PO_4_, and once with water. The extent of peptide library phosphorylation was determined by imaging the membrane with a phosphorimager (Molecular Dynamics).

### GST pulldowns.

For GST pulldown experiments, GST fusions were immobilized on glutathione-sepharose by incubating bead slurry with E. coli lysate containing GST protein at 4 °C for 15 min on a rotator. Beads were washed five times in PBS/KMD and incubated with His_6_-Xpo1 (5 μM final in 100 μl) at 4 °C for 15 min on a rotator. Beads were washed five times with 50 mM Tris, 150 mM NaCl, 0.1% Tween-20 (pH 7.5) (TBST), and bound protein was eluted by boiling in SDS-PAGE sample buffer for 10 min. Samples were separated by SDS-PAGE, stained with GelCode Blue (Pierce), and visualized on an Odyssey (Li-Cor) fluorescence scanner.

### Immunoprecipitation.

Cbk1-HA cells (225 OD_600_) grown to mid-logarithmic phase were lysed and immunoprecipitated as described previously [20]. Immunoprecipitates used in kinase assays were stored in yeast wash buffer (50 mM Tris [pH 7.5], 150 mM NaCl, 1 mM dithiothreitol, 0.5 mM leupeptin, and 1 μg/ml pepstatin) at 4 °C overnight.

### Kinase assays.

Kinase assays were performed as described previously [20]. Briefly, immunoprecipitated Cbk1-HA bound to protein G sepharose (Invitrogen) was divided and resuspended in kinase buffer (20 mM Tris [pH 6.8], 150 mM NaCl). A total of 10 μg of purified GST-Ace2 substrate was added to reaction buffer containing 5 mM MnCl_2_, 20 μM cold ATP, and 10 μCi/μl γ-^32^P-ATP. Reactions were incubated at room temperature for 1 h and quenched by addition of 5× SDS-PAGE sample buffer and 10 min incubation at 85 °C. Proteins were resolved on a 10% SDS-PAGE gel and transferred to a PVDF membrane (Pall). Fragment phosphorylation was evaluated using a Storm 860 Imager. Blots were incubated with anti-GST 1:10,000 (Sigma) and anti-HA 1:1,000 (12CA5, a gift of R. Lamb, Northwestern University, Evanston, Illinois) monoclonal antibodies overnight, then washed 3× with TBST and incubated with IRDye-800 goat anti-mouse secondary antibody (Rockland) (1:5,000) for 2 h at room temperature. Blots were imaged using an Odyssey scanner, and protein concentrations quantified using Odyssey software.

### Western blots and urea lysis.

Frozen Ace2-HA mutant pellets (5 OD_600_) were resuspended in 200 μl of 8M Urea, 50 mM HEPES (pH 7.4), and 200 μl of fine glass beads were added. Cells were lysed in a multivortexor at maximum speed for 5 min. A total of 20 μl of 25% SDS was added, and lysates were incubated at 65 °C for 5 min followed by centrifugation at 13.2*g* for 5 min. Cleared lysates were collected, and protein concentration was measured by Bradford assay. A total of 25 μg of lysate per lane was loaded onto an 8% SDS-PAGE gel and transferred to PVDF membranes. Blots were blotted with anti-HA 1:1,000 for 2 h at room temperature then stained with IRDye-800 conjugated goat anti-mouse antibodies (1:5,000) for 2 h. Images were collected and quantified using Odyssey software.

### Microscopy.

Cells were grown in synthetic medium to early log phase and pulse-labeled with rhodamine-conjugated concanavalin A (Vector) for 10 min, followed by 70 min of growth. Cells were imaged in synthetic dextrose medium at 25 °C with a 100×/1.45 NA oil-immersion objective using fluorescence/differential interference contrast microscopy with an Axiovert 200M (Carl Zeiss MicroImaging) and photographed with a Cascade II-512 camera (Photometrics). Contrast enhancement of images was performed using Openlab software. GFP *Z*-stacks were taken, and the brightest individual nuclei were measured for fluorescence intensity using Openlab software. For wild-type cells, only daughter nuclei were quantified; individual nuclei in mother–daughter pairs were scored for each Ace2 mutant. For cell separation quantification, cells were sonicated 2× 15 s prior to microscopy. Cells used for LMB experiments were grown in synthetic medium for 4 h, spun down, and resuspended in fresh medium containing 10 ng/μl LMB and grown for 30 min, then used for microscopy.

### Peptide synthesis.

Biotinylated peptides were synthesized by the MIT Biopolymers Laboratory: Biotin-Ahx-Y-SGTAIFGFL-GHNKTLSISSLQQSILNMSK (wild-type), Biotin-Ahx-Y-pSGTAIFGFLGHNKTLSISSLQQSILNMSK (pS122), Biotin-Ahx-Y-SGTAIFGFLGHNKTLpSISSLQQSILNMSK (pS137), Biotin-Ahx-Y-SGTAIVGFLGHNKTLSISSLQQSILNMSK (F127V), where Ahx denotes aminohexanoic acid. Synthesis of the dually phosphorylated (pS122/pS137) peptide was unsuccessful.

### Peptide binding.

For each biotin-peptide binding experiment, 10 nmol of peptide were dephosphorylated by treatment with 800 units of λ-phosphatase (New England Biolabs) at 30 °C for 60 min in a reaction volume of 100 μl (50 mM Tris, 100 mM NaCl, 0.1 mM EGTA, 2 mM DTT, 0.01% Brij 35, 2 mM MnCl_2_ [pH 7.5]); 900 μl of PBS/KMD, and 20 μl of streptavidin-sepharose (50% slurry in PBS/KMD; Amersham) were then added, and the suspension incubated at 4 °C for 15 min on a rotator. Non-phosphatase treated peptides were bound to steptavidin-sepharsoe similarly. Streptavidin beads with immobilized peptide were washed three times in PBS/KMD and incubated with His_6_-Crm1 (5 μM final in 100 μl) at 4 °C for 15 min on a rotator. Beads were washed five times with TBST, and bound protein was eluted by boiling in SDS-PAGE sample buffer for 10 min. Samples were separated by SDS-PAGE, stained with GelCode Blue, and visualized on an Odyssey fluorescence scanner.

### RT quantitative PCR.

RNA extracted from 5 OD_600_ of cells grown to mid-logarithmic phase was prepared as described [40]. A total of 4 μg of RNA was reverse transcribed using 7 μM T120V primer and reverse transcriptase at 42 °C, for 1 h; 50 ng of total product was used in subsequent quantitative PCR reactions using *CTS1*, *DSE1*, and *ACT1* primers. Standard curves for each primer were generated using serial dilutions of yeast genomic DNA and linear regression analysis of cycle threshold (Ct) values. Quantification of cDNA template concentrations were calculated using the standard curve for each primer.

### Chromatin immunoprecipitation.

Cells (70 OD_600_) grown to mid-logarithmic phase were incubated with 1% formaldehyde at room temperature for 30 min then filtered and washed with 100 mM Tris (pH 7.0) and flash frozen with liquid nitrogen. Pellets were lysed in ChIP lysis buffer (50 mM HEPES [pH 7.5], 140 mM NaCl, 1 mM EDTA, 1% Triton-X-100, 0.1% NaDOC, 0.5 mM pepstatin, and 1 μg/ml leupeptin) by bead beating in a multivortexor. Lysates were cleared by centrifugation at 7,000 rpm for 10 min, and pellets were resuspended in ChIP lysis buffer (without detergent) and sonicated, setting 7, 10× 10 s with 10 s rests. One percent Triton-X-100 and 0.1% NaDOC were added and centrifuged 10 min at 14,000 rpm. Supernatant was collected and incubated with 2 μg of anti-HA on ice for 30 min. Protein-G Dynabeads (Dynal Biotech ASA) were incubated with lysate 1 h, 4 °C, rotating. Input samples were collected and incubated with elution buffer (50 mM Tris [pH 8], 10 mM EDTA, 1% SDS), 65 °C overnight. Beads were washed 10× with ChIP lysis buffer and eluted with elution buffer at 65 °C, 15 min, then washed with 50 mM Tris (pH 8), 10 mM EDTA, 0.67% SDS. Eluates and washes were pooled, and incubated 65 °C overnight. DNA was purified using QIAquick PCR Purification Kit (Qiagen) and used in Q-PCR reactions with primers for *DSE1*, *SCW11*, and *ACT1* loci. Standard curves and quantification were performed as described above.

## Supporting Information

Figure S1 Phosphorylation of Individually Mutated Cbk1 Phosphoacceptor Sites In vitro phosphorylation of Ace2 N-terminal domain GST fusions by immunoprecipitated Cbk1-HA. Mutation of residues S113, S122, and S137 to Ala abolishes phosphorylation as does mutation of H117 to Ala (lanes 4 and 5) as shown in Figure 2C. Cbk1 still efficiently phosphorylates the GST-Ace2 fragment when phosphoacceptor sites are singly mutated to Ala (lanes 6–8).
(309 KB PDF)

Figure S2 Proteins Levels of *ace2* Alleles C-terminal fusions to an HA tag were constructed for all *ace2* alleles. Levels were assessed by immunoblot with anti-HA and compared with levels for wild-type Ace2-HA fusion. Twenty-five micrograms of cell lysate were loaded per lane.
(205 KB PDF)

Figure S3 Quantification of Cell Separation in *ace2* Mutant Strains The number of connected cells were quantified in three independent trials (*n* = 200) for all Ace2 mutant alleles, and averages are shown. Wild-type and *ace2Δ* strains are shown for comparison. Clumps of cells were binned into groups according to the number of cells in each clump. Error bars show the standard deviation of the average of three trials.
(696 KB PDF)

Figure S4 Quantitative RT-PCR Analysis of Ace2 Target Genes RNA was extracted from all Ace2 mutant alleles as well as wild-type, *ace2Δ*, and *cbk1Δ* strains and reverse transcribed to obtain cDNA. The concentration of cDNA for two Ace2 target genes (*CTS1* and *DSE1*) was quantified relative to *ACT1* mRNA by real-time quantitative PCR. Three independent trials were performed, and averages are shown. Error bars denote standard deviations of the average of three trials.
(164 KB PDF)

Figure S5 Mutations in Ace2 NES Reduce Binding to Crm1 In Vitro Single phosphomimic S122D and S137D mutations in Ace2 reduce its binding to Crm1 in an in vitro GST pulldown assay. Binding is undetectable in the double mutant, *ace2-S122D/S137D*. A F127V mutation also significantly reduces binding to Crm1.
(191 KB PDF)

## Abbreviations

CDK: cyclin-dependent kinase
ChIP: chromatin immunoprecipitation
GMC: ganglion mother cells
LMB: leptomycin B
NES: nuclear export sequence
RAM: Regulation of Ace2 and Morphogenesis
RT-PCR: real-time polymerase chain reaction
