# Supplementary material for: The NDR/LATS Family Kinase Cbk1 Directly Controls Transcriptional Asymmetry
Source: PLoS Biol. 2008 Aug 19;6(8):e203. doi: 10.1371/journal.pbio.0060203 (PMC2517623; doi:10.1371/journal.pbio.0060203)
Supplement: Supplementary file 1 [file pbio.0060203.sg001.pdf]

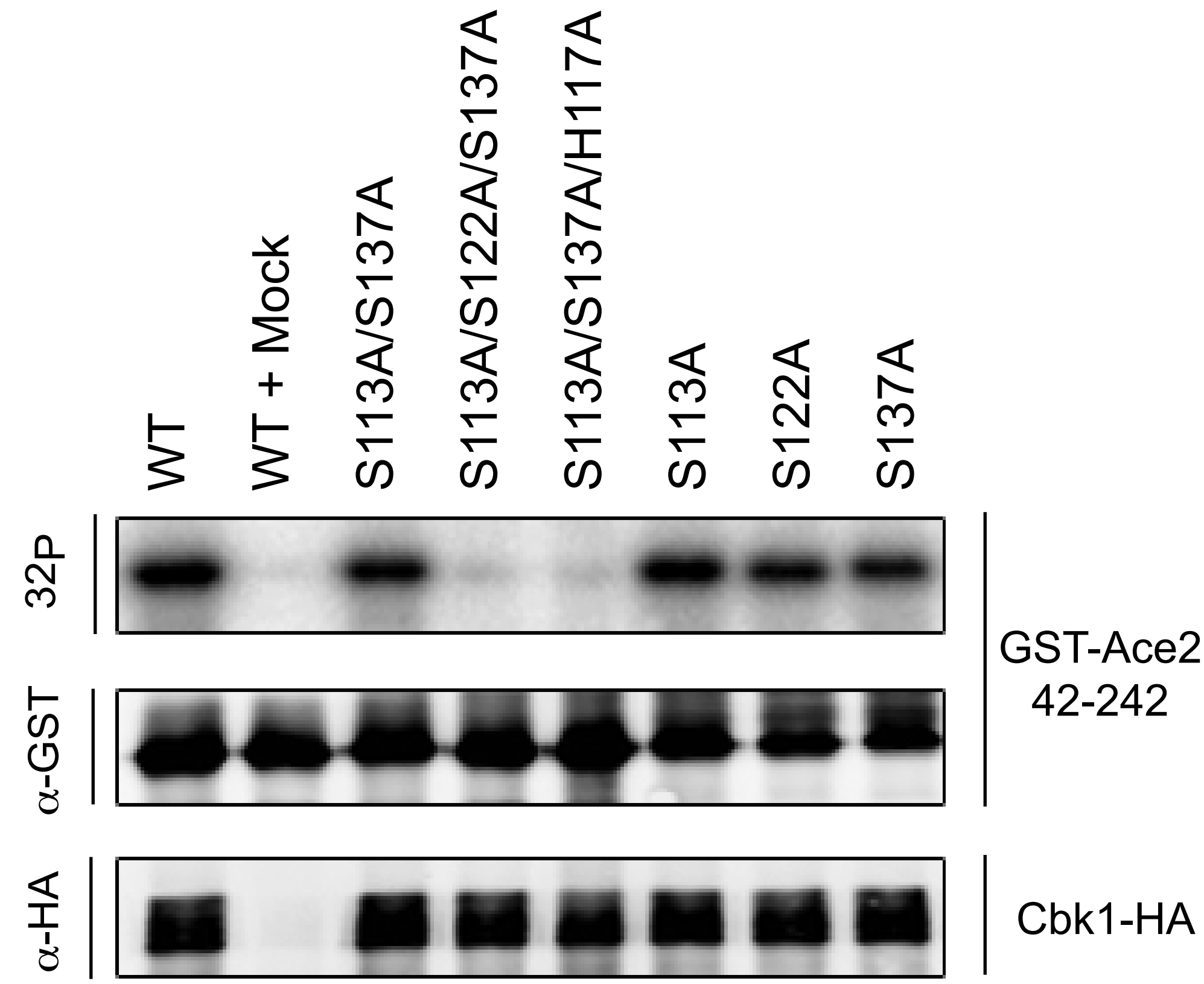
WT
WT + Mock
S113A/S137A
S113A/S122A/S137A
S113A/S137A/H117A
S113A
S122A
S137A
32P
α-GST
α-HA
GST-Ace2
42-242
Cbk1-HA
