# Supplementary material for: The NDR/LATS Family Kinase Cbk1 Directly Controls Transcriptional Asymmetry
Source: PLoS Biol. 2008 Aug 19;6(8):e203. doi: 10.1371/journal.pbio.0060203 (PMC2517623; doi:10.1371/journal.pbio.0060203)
Supplement: Supplementary file 2 [file pbio.0060203.sg002.pdf]

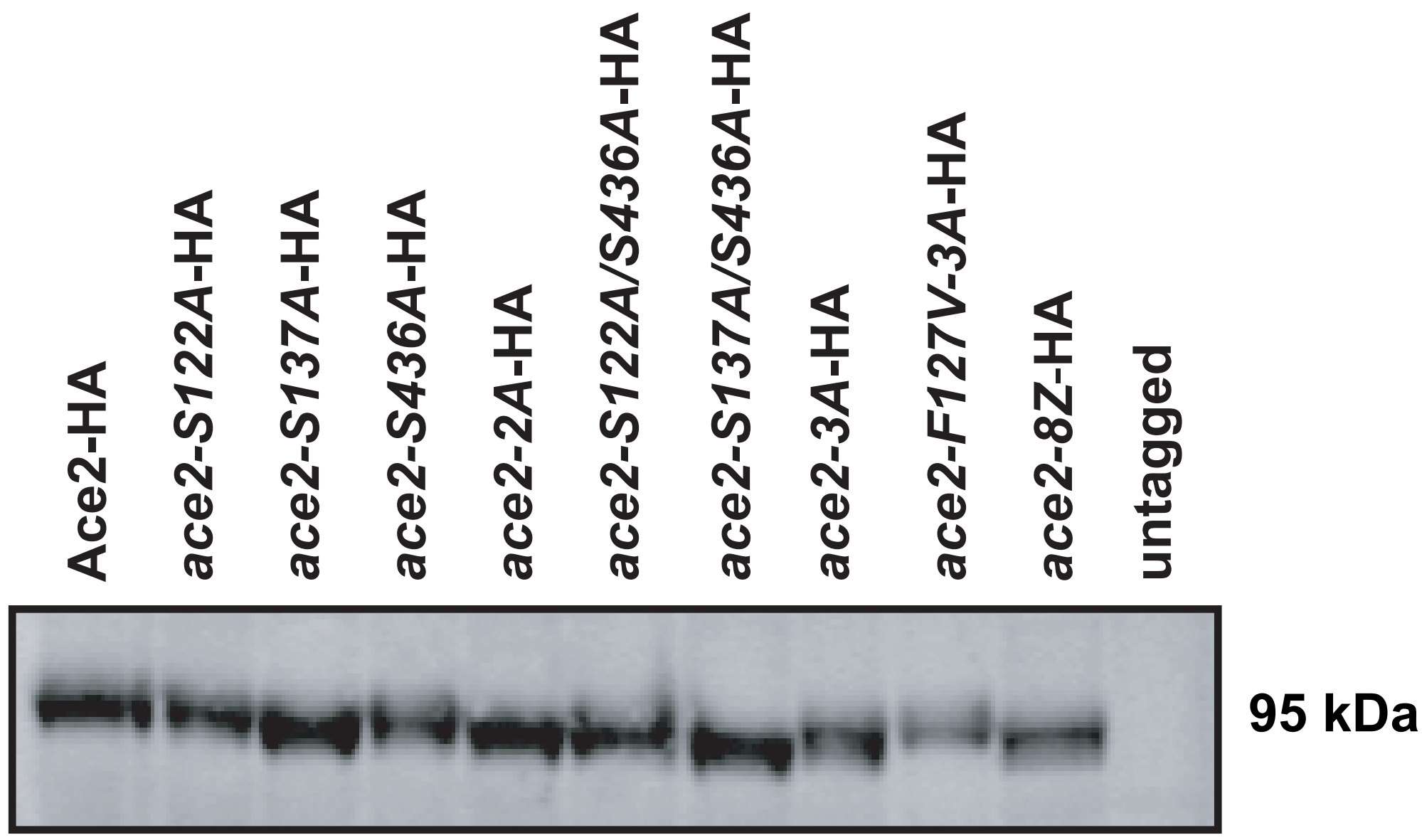
Ace2-HA
ace2-S122A-HA
ace2-S137A-HA
ace2-S436A-HA
ace2-2A-HA
ace2-S122A/S436A-HA
ace2-S137A/S436A-HA
ace2-3A-HA
ace2-F127V-3A-HA
ace2-8Z-HA
untagged
95 kDa
