# Supplementary material for: The NDR/LATS Family Kinase Cbk1 Directly Controls Transcriptional Asymmetry
Source: PLoS Biol. 2008 Aug 19;6(8):e203. doi: 10.1371/journal.pbio.0060203 (PMC2517623; doi:10.1371/journal.pbio.0060203)
Supplement: Supplementary file 3 [file pbio.0060203.sg003.pdf]

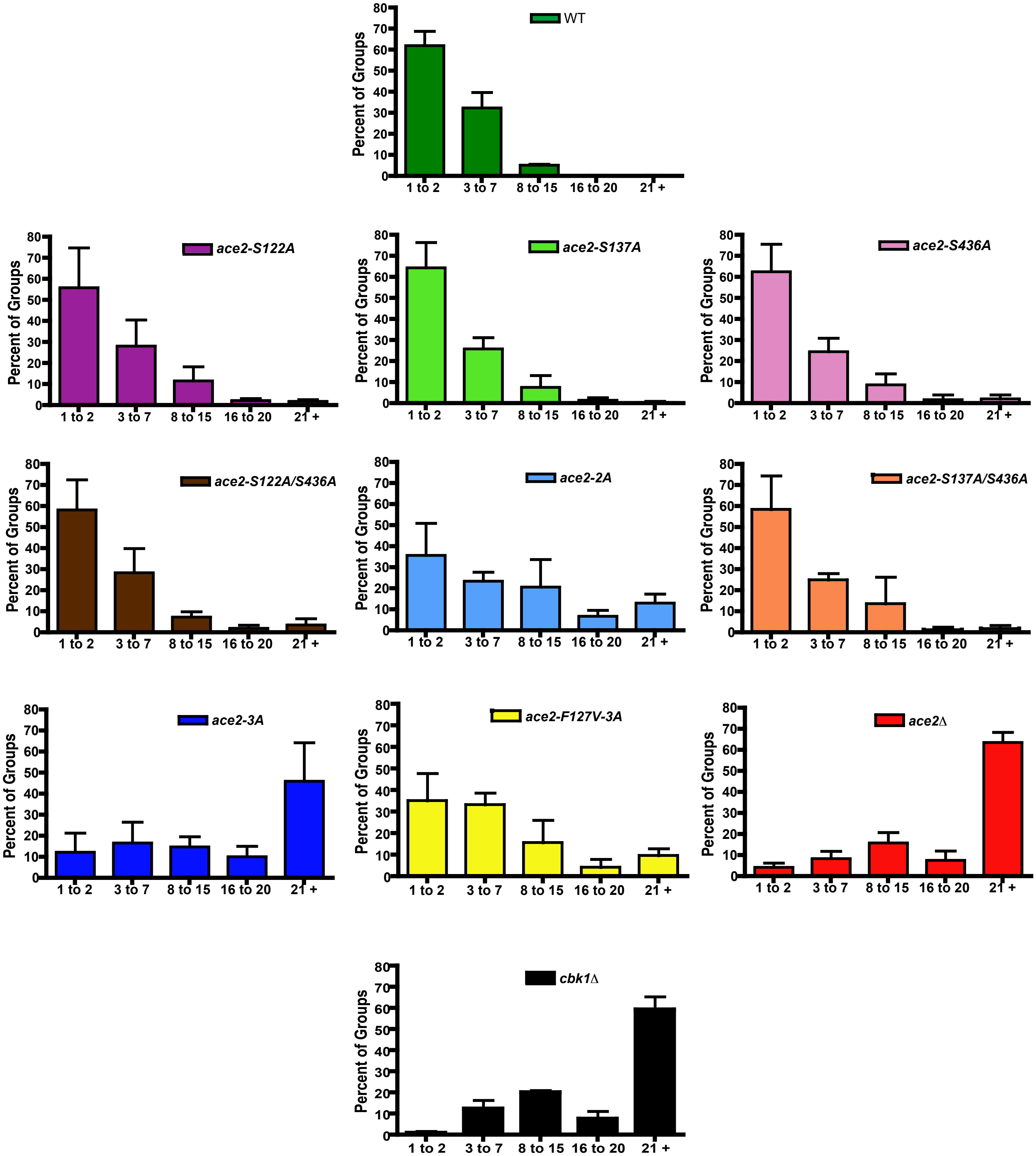

WT
ace2-S122A
ace2-S137A
ace2-S436A
ace2-S122A/S436A
ace2-2A
ace2-S137A/S436A
ace2-3A
ace2-F127V-3A
ace2Δ
cbk1Δ
Percent of Groups
0
10
20
30
40
50
60
70
80
1 to 2
3 to 7
8 to 15
16 to 20
21 +
