# Supplementary material for: The NDR/LATS Family Kinase Cbk1 Directly Controls Transcriptional Asymmetry
Source: PLoS Biol. 2008 Aug 19;6(8):e203. doi: 10.1371/journal.pbio.0060203 (PMC2517623; doi:10.1371/journal.pbio.0060203)
Supplement: Supplementary file 4 [file pbio.0060203.sg004.pdf]

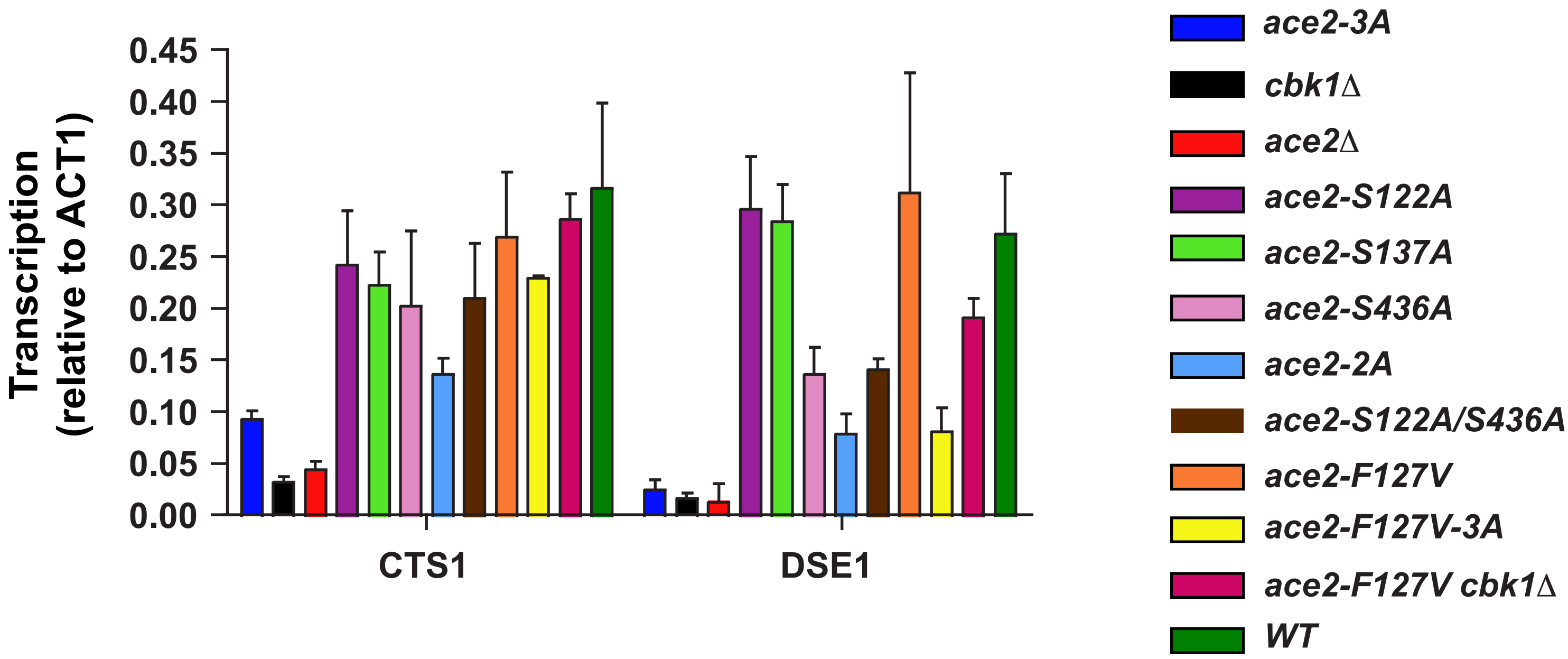
Transcription
(relative to ACT1)
0.45
0.40
0.35
0.30
0.25
0.20
0.15
0.10
0.05
0.00
CTS1
DSE1
ace2-3A
cbk1Δ
ace2Δ
ace2-S122A
ace2-S137A
ace2-S436A
ace2-2A
ace2-S122A/S436A
ace2-F127V
ace2-F127V-3A
ace2-F127V cbk1Δ
WT
