# Supplementary material for: The NDR/LATS Family Kinase Cbk1 Directly Controls Transcriptional Asymmetry
Source: PLoS Biol. 2008 Aug 19;6(8):e203. doi: 10.1371/journal.pbio.0060203 (PMC2517623; doi:10.1371/journal.pbio.0060203)
Supplement: Supplementary file 5 [file pbio.0060203.sg005.pdf]

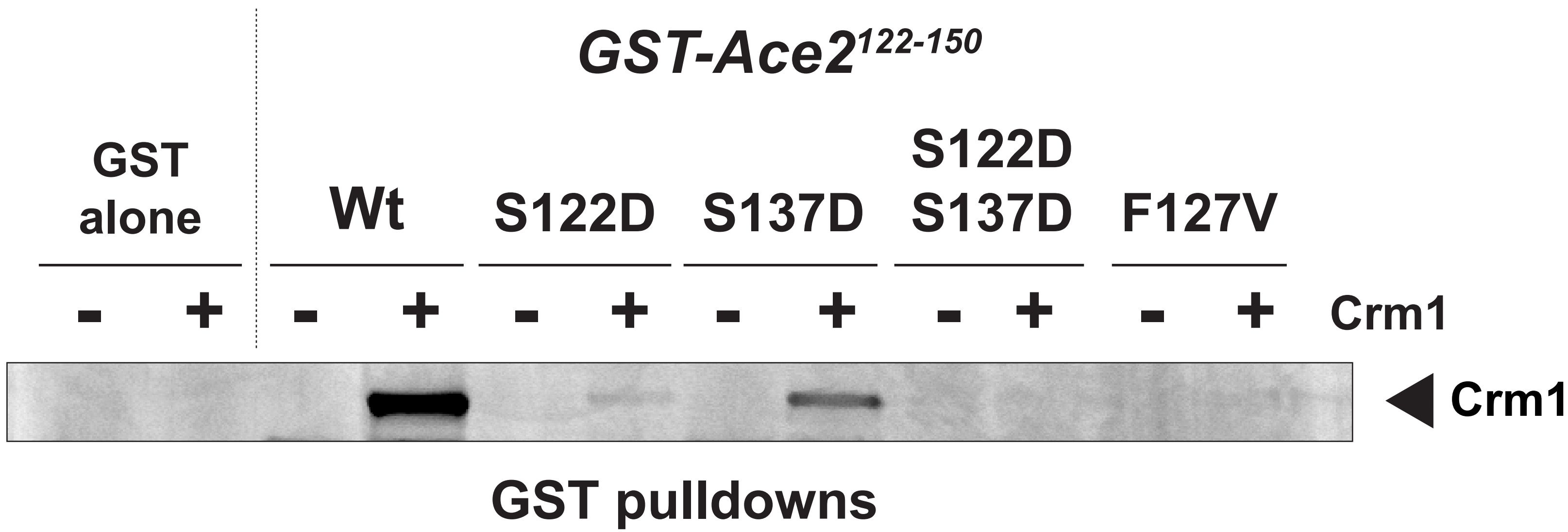
GST-Ace2^122-150
GST alone
Wt
S122D
S137D
S122D S137D
F127V
- + - + - + - + - + - +
Crm1
Crm1
GST pulldowns
